# Gene target specificity of the Super Elongation Complex (SEC) family: how HIV-1 Tat employs selected SEC members to activate viral transcription

**DOI:** 10.1093/nar/gkv541

**Published:** 2015-05-24

**Authors:** Huasong Lu, Zichong Li, Wei Zhang, Ursula Schulze-Gahmen, Yuhua Xue, Qiang Zhou

**Affiliations:** 1 Innovation Center of Cell Signaling Network, School of Pharmaceutical Sciences, Xiamen University, Xiamen 361005, Fujian, China; 2 Department of Molecular and Cell Biology, University of California, Berkeley, Berkeley, CA 94720, USA

## Abstract

The AF4/FMR2 proteins AFF1 and AFF4 act as a scaffold to assemble the Super Elongation Complex (SEC) that strongly activates transcriptional elongation of HIV-1 and cellular genes. Although they can dimerize, it is unclear whether the dimers exist and function within a SEC *in vivo*. Furthermore, it is unknown whether AFF1 and AFF4 function similarly in mediating SEC-dependent activation of diverse genes. Providing answers to these questions, our current study shows that AFF1 and AFF4 reside in separate SECs that display largely distinct gene target specificities. While the AFF1-SEC is more potent in supporting HIV-1 transactivation by the viral Tat protein, the AFF4-SEC is more important for HSP70 induction upon heat shock. The functional difference between AFF1 and AFF4 in Tat-transactivation has been traced to a single amino acid variation between the two proteins, which causes them to enhance the affinity of Tat for P-TEFb, a key SEC component, with different efficiency. Finally, genome-wide analysis confirms that the genes regulated by AFF1-SEC and AFF4-SEC are largely non-overlapping and perform distinct functions. Thus, the SEC represents a family of related complexes that exist to increase the regulatory diversity and gene control options during transactivation of diverse cellular and viral genes.

## INTRODUCTION

The elongation stage of RNA polymerase (Pol) II transcription plays an important role in controlling the expression of many cellular and viral genes (1). The activation of Pol II elongation along the integrated HIV-1 proviral DNA by the viral encoded Tat protein is absolutely essential for productive HIV-1 infection (2,3). This process has long been used as a model system for studying the mechanism and factors that control Pol II elongation. Pioneering studies demonstrate that Tat transactivates HIV-1 by recruiting the human Positive Transcription Elongation Factor b (P-TEFb) to the viral promoter through forming a complex on the TAR RNA stem-loop structure that is located at the 5′ end of all nascent viral transcripts (4). Composed of CDK9 and its regulatory partner cyclin T1 (CycT1) or T2a/2b, P-TEFb releases Pol II from promoter-proximal pausing through phosphorylating two negative elongation factors NELF and DSIF to antagonize their inhibitory actions (2,3). In addition, P-TEFb also phosphorylates the carboxy-terminal domain (CTD) of the largest subunit of Pol II, which in turn promotes co-transcriptional mRNA processing (1).

Although P-TEFb is necessary for Tat activation of HIV-1 transcription, it is insufficient to support maximal Tat function (5,6). Affinity purifications followed by proteomics have led to the identification of the Super Elongation Complex (SEC), in which P-TEFb is an integral component, as the native form of human co-factor recruited by Tat to the viral promoter for full HIV-1 transactivation (7,8). Besides P-TEFb, the SEC also contains ELL1 or ELL2, a member of the ELL family of elongation stimulatory factors, which can directly suppress Pol II pausing and synergize with P-TEFb to greatly promote Tat-transactivation (7).

Within the SEC, the AF4/FMR2 family proteins AFF1 and AFF4 function as a central scaffold that uses short hydrophobic regions distributed along the highly flexible axis to interact with other subunits and assemble the complete SEC (9,10). The recently solved crystal structure containing the CycT1-binding sequence (CBS) of AFF4 in complex with human P-TEFb and HIV-1 Tat has revealed direct contacts between AFF4 and Tat on the surface of CycT1 (11,12). The highly cooperative nature of the interactions among these three SEC subunits underlies the AFF4-mediated increase of the affinity of Tat for P-TEFb.

At about the same time when the SEC was identified as a specific Tat co-factor, the same complex was also found to be hijacked by several MLL (mixed lineage leukemia) fusion proteins that are created by chromosomal translocations to cause aberrant activation of key MLL-target genes and acute childhood leukemia (13–15). Notably, the SEC subunits AFF1 and AFF4 are among the most common translocation partners of MLL during leukemogenesis (16). It has been proposed that the oncogenic potential of MLL-AFF1 and MLL-AFF4 is conferred by their associations with endogenous active SEC complex through dimerization mediated by the C-terminal homology domains (CHDs) of AFF1/4 (15). However, whether in the absence of the fusions to MLL, the dimers formed by native AFF1/4 can also reside and function within a single SEC complex *in vivo* has not been officially examined. Furthermore, despite their sequence similarity, it remains to be determined whether AFF1 and AFF4 function similarly in mediating the SEC-dependent activation of transcription of diverse genes.

The current study seeks to answers these questions, and our data show that AFF1 and AFF4 reside in separate SEC complexes that display largely distinct activator/gene-target specificities. Between the two homologous SEC complexes, the AFF1-SEC is more potent in mediating Tat-transactivation, whereas the AFF4-SEC is more important for proper HSP70 induction in response to heat shock. The functional difference between AFF1 and AFF4 in supporting Tat-transactivation has been traced to a single amino acid variation in the CBS region between the two AFF proteins, which causes them to enhance the affinity of Tat for CycT1 with different efficiency. Finally, genome-wide analysis has also confirmed that the genes regulated by the AFF1- and AFF4-containing SECs are largely non-overlapping and participate in distinct biological functions/pathways. Together, our data support the model that the SEC represents a family of related complexes that exist to increase the regulatory diversity and gene control options during transcriptional activation of diverse cellular and viral genes.

## MATERIALS AND METHODS

### Antibodies

Polyclonal antibodies against AFF1 (A302-344A), ELL1 (A301-645A), ELL2 (A302-505A), ENL (A302-268A) and AF9 (A300-595A) were purchased from Bethyl Laboratories. Anti-AFF4 (ab57077) antibody was purchased from Abcam. The monoclonal antibodies against Flag (M2) and HA (3F10) were from Sigma-Aldrich and Roche, respectively. The antibodies against CDK9, LARP7 and HEXIM1 were generated in our own laboratory and have been described previously (17,18).

### Generation of 293-F9H4 cells that stably express Flag-tagged CDK9 and inducibly express HA-tagged AFF4

The T-RExTM-293 (Invitrogen)-based cell line that stably expresses CDK9-F and confers puromycin-resistance ((7), renamed 293-F9) was used to generate 293-F9H4 stable cell line. AFF4 cDNA was cloned into pCDNAh/TO vector with an HA tag at the C-terminus. The expression plasmid was stably transfected into 293-F9 cells and selected with hygromycin for two weeks. Individual cell colonies were picked and screened for the inducible expression of AFF4-HA upon doxycycline treatment (1 μg/ml) for 48 h. For tandem affinity-purification of the SEC containing both CDK9-F and AFF4-HA within a single complex, the procedure described previously (7) was used.

### Quantitative PCR

The reactions were performed with Applied Biosystem 7300 Real-Time PCR System and DyNAmo HS SYBR Green qPCR reagents according to the manufacturers’ instructions. PCR primers were designed with Integrated DNA Technologies’ Primer Quest. The PCR conditions include an initial denaturing step at 92°C for 2 min and then 40 (for qRT-PCR) or 50 (for ChIP-PCR) cycles of amplification. Each cycle consists of a 92°C segment of 30 s, a 57°C segment of 30 s and then a 68°C segment of 30 s. For ChIP-PCR, threshold values (Ct) were calculated and normalized to the input. For qRT-PCR, the values were normalized to those of GAPDH to obtain the relative folds of induction. All reactions were run in triplicates.

### Chromatin immunoprecipitation assay

The chromatin immunoprecipitation (ChIP) assay was performed as described (7) with some modifications. Briefly, HeLa cells were incubated at 42°C for 2 h for heat-shock and then cross-linked with 1% formaldehyde for 10 min. Cross-linking was quenched by the addition of glycine (0.125 M for 5 min). Fixed cells were collected and re-suspended in SDS lysis buffer (1% SDS, 10 mM EDTA, 50 mM Tris, pH 8.1) and fragmented using a Covaris-S2 sonicator (Covaris, Inc., Woburn, MA) for a total processing time of 25 min (30 s on and 30 s off). Sonicated lysates equivalent to 2×10^6^ cells were incubated overnight with 3 μg specific antibodies per reaction, and the purified products were analyzed by qPCR. All signals were normalized to the input DNA, and signals generated by non-specific IgG in control immunoprecipitations were subtracted from the signals obtained with specific antibodies.

### RNA-seq analysis in AFF1/4 knockdown cells

Total RNA extracted from each knockdown (KD) cells were depleted of rRNA with Ribo-zero (Illumina) and converted into multiplexed libraries using mRNA-seq Trueseq Kit following the manufacturer's instructions (Illumina). The libraries were then multiplexed and sequenced on Illumina HiSeq 2000 sequencer. All sequencing reads were aligned to the human reference genome (UCSC hg19 release) and RefSeq reference transcriptome (ftp://ftp.ncbi.nih.gov/refseq) using TopHat version 2.0.11 (19). Cufflinks version 2.2.1 (20) was used to quantify the mRNA abundance for each gene (Fragments Per Kilobase of exon model per Million mapped fragments, referred to as FPKM). The following non-default options were used with cufflinks: frag-bias-correct and multi-read-correct. RankProd (21) was applied to perform differential expression analysis between AFF1/4 KD and GFP KD samples. RNA-seq data have been deposited at GEO database with the accession number GSE69021.

### Gene ontology enrichment analysis

Gene ontology (GO) enrichment analysis was performed using DAVID Bioinformatic Resources (22).

### Constructing the network

Human functional protein interaction network (23) was used as a template to construct the sub-networks among the DEGs induced by AFF1 or AFF4 KD. The network template consists of manually curated interactions (MSKCC cancer cell map; http://cancer.cellmap.org); NCI-Nature pathway interaction database (http://pid.nci.nih.gov); KEGG (24); BioCarta (http://www.biocarta.com/genes/index.asp); Reactome (25); TRED (26); and PANTHER (27), and also predicted interactions derived from non-curated sources. Structurally dense regions in the networks were identified by the Markov Clustering (MCL) algorithm (http://www.micans.org/mcl/) with the granularity parameter set to 2.0.

## RESULTS

### AFF1 and AFF4 form hetero- but not homodimers and the heterodimerization is not required for SEC assembly

**Figure 1.**
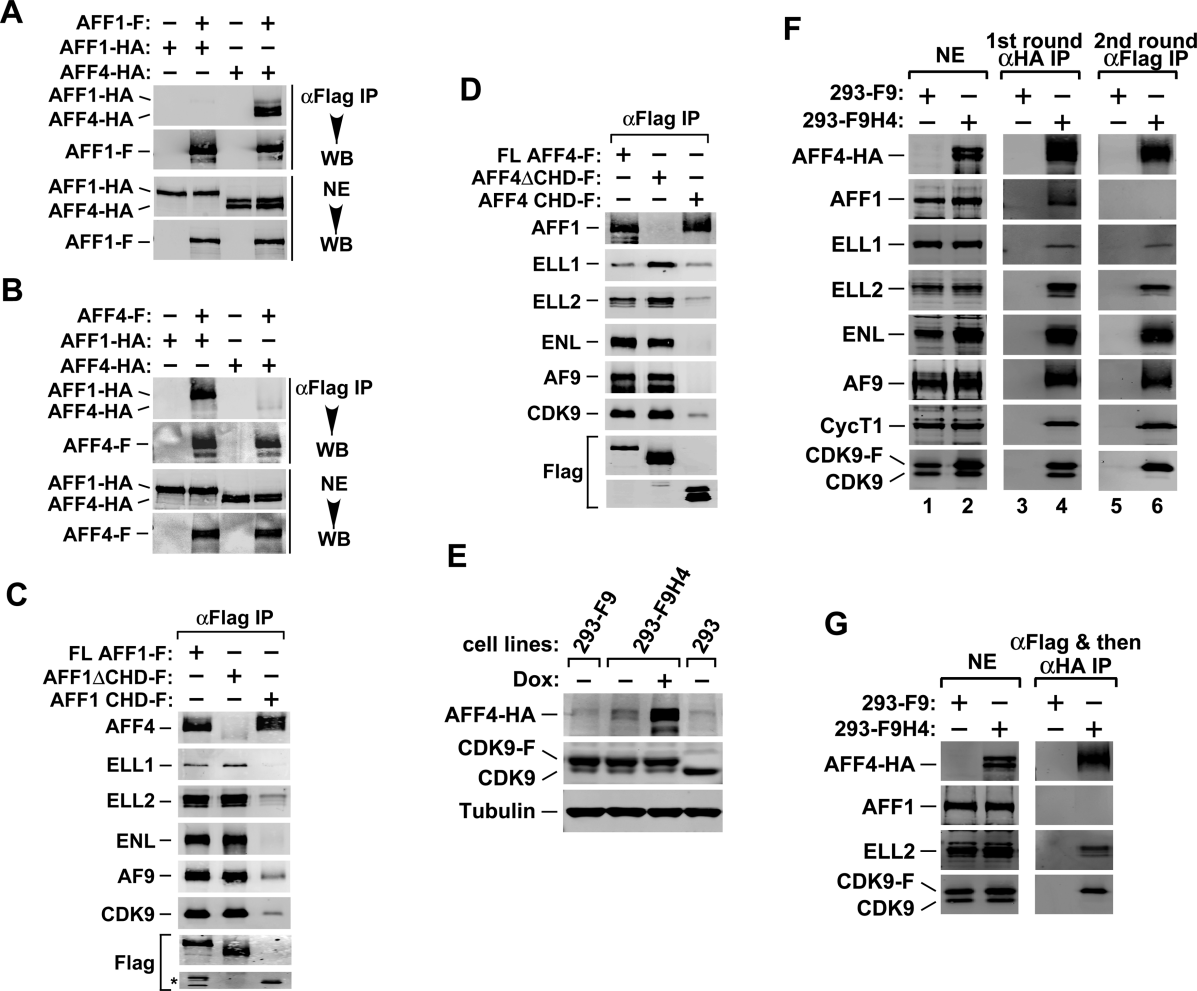
AFF1 and AFF4 reside in different SECs. (**A,B**) Nuclear extracts (NEs) derived from HeLa cells co-transfected with the indicated cDNA constructs were subject to anti-Flag immunoprecipitation (IP). The precipitates and NEs were examined by western blotting (WB) for the indicated proteins. (**C,D**) HeLa cells were transfected with plasmid vectors expressing the indicated Flag-tagged WT AFF1 (C), AFF4 (D) or their mutant derivatives. Anti-Flag immunoprecipitates from NE were analyzed by WB for the various proteins as marked on the left, with the star (*) in C denotes the position of two minor truncated AFF1 bands. (**E**) Whole cell extracts of the indicated HEK293-based cell lines were analyzed by WB. Both 293-F9 and 293-F9H4 stably expressed CDK9-F, while the latter also expressed AFF4-HA upon induction by doxycycline (Dox). (**F,G**) CDK9-F, AFF4-HA and their associated factors were isolated through sequential immunoprecipitations (anti-HA and then anti-Flag in F; anti-Flag and then anti-HA in G) from NEs of 293-F9H4 cells upon induction of AFF4-HA expression. The immunoprecipitates were analyzed by WB for the proteins indicated. NEs of 293-F9 cells were used in parallel procedures as controls.

We began our investigation by examining the dimer formation between AFF1 and AFF4 when they are not fused to MLL. To this end, differentially epitope-tagged AFF1 and AFF4 were co-expressed in HeLa cells to assess their interaction with oneself and each other. Surprisingly, FLAG-tagged AFF1 (AFF1-F) co-immunoprecipitated efficiently with HA-tagged AFF4 (AFF4-HA) but not AFF1-HA (Figure 1A). Likewise, AFF4-F readily pulled down AFF1-HA but not AFF4-HA (Figure 1B). Thus, AFF1 and AFF4 formed mostly hetero- but not homodimers between them.

To determine the functional significance of AFF1-AFF4 heterodimerization for the SEC assembly, we performed co-immunoprecipitation (co-IP) experiments to examine the abilities of AFF1/4 truncation mutants to interact with other SEC components as well as their respective heterodimerization partners. While full length (FL) AFF1 (aa 1-1210) readily co-precipitated with ELL1, ELL2, ENL, AF9, CDK9 and its heterodimer partner AFF4, deletion of the CHD (Caa 903-1210) in AFF1 (AFF1ΔCHD) completely disrupted the binding to AFF4 but maintained normal interactions with the other SEC subunits (Figure 1C). On the other hand, compared to FL AFF1, the isolated CHD associated with a similar level of AFF4 but showed drastically reduced binding to the other SEC components. Nearly identical results were also obtained with the AFF4 truncation mutants (Figure 1D; CHD of AFF4: aa 848-1167). Together, these results suggest that the AFF1-AFF4 heterodimerization did not play a significant role in the SEC assembly.

### AFF1 and AFF4 reside in separate SECs

Consistent with the above observations, *in vitro* binding studies performed in the Roeder laboratory have shown a mutually exclusive existence of AFF1 and AFF4 in reconstituted SEC complexes (28). To confirm that those two proteins also do not exist in a single SEC complex *in vivo*, we established an HEK293-based cell line called 293-F9H4 that stably expresses Flag-tagged CDK9 and inducibly expresses HA-tagged AFF4 (Figure 1E) and performed tandem affinity-purification of the SEC that contained both CDK9-F and AFF4-HA within a single complex. The parental HEK293 and its derivative 293-F9 that only stably expresses CDK9-F were used as controls (Figure 1E).

Western blotting analysis indicates that after the first round IP with anti-HA mAb, all the known SEC components as well as AFF1 were readily detected in precipitates derived from nuclear extracts (NEs) of 293-F9H4 but not 293-F9 cells (Figure 1F, lane 4). This result suggests that the IP was specific, and more importantly, that AFF4-HA became efficiently incorporated into both the SEC and the AFF1-AFF4 heterodimer *in vivo*. However, after the second round IP with anti-Flag mAb, which precipitated all the factors present in the same complex with *both* CDK9-F and AFF4-HA, only AFF4 and the signature SEC subunits ELL1, ELL2, ENL, AF9, CycT1 and CDK9 but not AFF1 were detected in the precipitates (lane 6). The same conclusion was also reached when we reversed the order of the tandem affinity-purification by first performing anti-Flag and then anti-HA IP in NE of 293-F9H4 and 293-F9 cells (Figure 1G). The absence of AFF1 in the tandem affinity-purified AFF4-containing SEC under both conditions indicates that AFF1 and AFF4 resided in separate SECs and that the AFF1-AFF4 heterodimer existed mostly outside of an SEC.

### AFF1 is more potent than AFF4 in supporting Tat-dependent HIV-1 transcription

**Figure 2.**
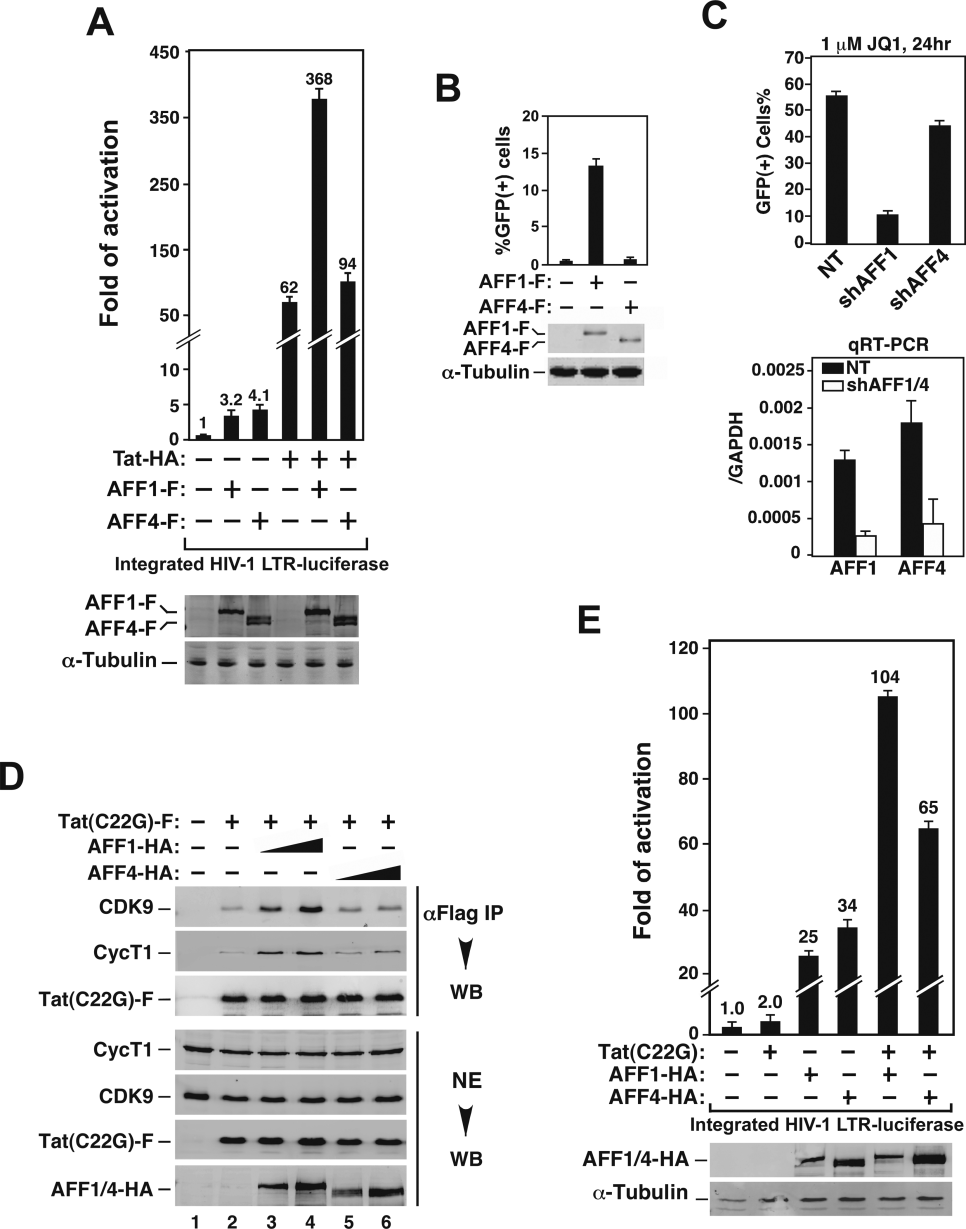
AFF1 supports Tat-dependent HIV-1 transcription more efficiently and increases the affinity of Tat for P-TEFb more potently than does AFF4. (**A**) Luciferase activities were measured in extracts of NH1 cells containing the integrated HIV-1 LTR-luciferase reporter gene and transfected with the Tat-HA- and/or AFF1/4-F-expressing construct as labeled. The activity in cells transfected with only the empty vectors was artificially set to 1. The error bars represent mean ± SD from three independent measurements. The cellular AFF1/4-F levels were determined by anti-Flag western blotting (WB). (**B**) J-Lat A2 cells were nucleofected with the AFF1/4-F-expressing construct as labeled. The induction of GFP expression was measured by flow cytometry and expressed as percentages of GFP(+) cells of the entire population. Cellular AFF1/4-F levels were detected as in A. (**C**) Top panel: 2D10 cells were nucleofected with plasmids expressing a non-targeting shRNA (NT) or a specific shRNA targeting AFF1 or AFF4. The activation of the HIV-1 LTR-driven GFP expression was analyzed as in B. Bottom panel: the knockdown efficiency of the shRNAs was determined by qRT-PCR, with the mRNA ratios of AFF1/4 over GAPDH displayed. (**D**) Nuclear extracts (NEs) from HeLa cells co-transfected with the indicated expression constructs were subjected to anti-Flag immunoprecipitation (IP). The precipitates and NEs were analyzed by WB. (**E**) Luciferase activities expressed from the integrated HIV-1 LTR in NH1 cells that were transfected with the Tat (C22G)- and/or AFF1/4-HA-expressing construct were measured and analyzed as in A.

As functional redundancy is often found among homologous proteins, we next asked whether the AFF1- and AFF4-containing SECs display similar or different functions in mediating transcription. Because the activation of HIV-1 transcription by Tat has been demonstrated as strongly SEC-dependent (5,7), we first compared the effects of the AFF1- and AFF4-SEC on Tat activation of luciferase expression from the integrated HIV-1 LTR in the HeLa-based NH1 cells (29). Data in Figure 2A indicate that although the ectopically expressed AFF1 and AFF4 were both able to enhance the Tat-activated as well as basal HIV-1 LTR activity, they did it with different efficiency. When normalized for expression level, AFF1 displayed markedly stronger ability to promote Tat-dependent HIV-1 transcription than did AFF4 on a per-molecule basis. As for the stimulation of basal HIV transcription, however, AFF4 was slightly more active than AFF1 (4.1- versus 3.2-fold, Figure 2A). In addition to NH1 cells, the impact of AFF1/4 ectopic expression on HIV-1 transcription was also determined in Jurkat-based J-Lat A2 cells, which contains an integrated 5′ LTR-Tat-IRES-EGFP-3′ LTR cassette and is a popular model for studying post-integrative HIV latency (30). Again, AFF1 was found to stimulate the Tat-dependent HIV-1 LTR-driven GFP expression much more efficiently than did AFF4 in this system (Figure 2B).

To investigate the roles of the AFF1- and AFF4-SEC in HIV-1 transcription from a different angle, we employed short hairpin (sh)RNAs to KD AFF1/4's expression and examined the impact on JQ1 activation of HIV-1 latency in 2D10 cells, another well-studied latency model created in Jurkat T cells (31). Recent data indicate that BRD4 acts as a direct competitor of HIV-1 Tat for binding to the P-TEFb component of the SEC (29,32–34) and that the BET bromodomain inhibitor JQ1 antagonizes BRD4's inhibitive effect to activate latent HIV (29,35).

In 2D10 cells expressing target-specific shRNAs, analysis by qRT-PCR reveals that the KD efficiency was very similar between AFF1 and AFF4 (Figure 2C, right panel). Under such conditions, the activation of the LTR-driven GFP expression by JQ1 was significantly reduced in the AFF1 KD cells but only weakly diminished in the AFF4 KD cells (Figure 2C, left panel). Together, these results support the notion that the AFF1-SEC is more potent than the AFF4-SEC in supporting Tat-dependent HIV-1 transcription and latency activation.

### AFF1 increases the affinity of Tat for P-TEFb more potently than does AFF4

What could be the molecular basis underlying the functional difference between AFF1 and AFF4 in their support of Tat-transactivation? Recent structural studies indicate that AFF4 promotes the Tat-CycT1 interaction by creating a deeper Tat-binding pocket than that formed by CycT1 alone (11). In light of this revelation, we decided to investigate whether AFF1 may behave differently in this regard, which can potentially explain the functional difference between the two AFF proteins.

To determine the impact of AFF1/4 on Tat-P-TEFb interaction, we used the C22G mutant Tat, which lacks an essential Cys-zinc bridge required for proper folding and is thus highly sensitive to the AFF-mediated increase in affinity for P-TEFb (5), in the binding assay. While the introduction of increasing amounts of AFF1 into cells allowed Tat(C22G)-F to pull down more CDK9 and CycT1 in a dosage-dependent manner (Figure 2D, compare lanes 3 & 4 with 1 & 2), introduction of comparable amounts of AFF4 produced a significantly smaller effect (lanes 5 & 6).

Consistent with their different abilities to promote the Tat-P-TEFb interaction, AFF1 turned Tat(C22G) into a powerful activator of the HIV-1 LTR (from 2.0- to 104-fold) when the two were expressed together (Figure 2E). In comparison, the co-expression with AFF4 caused Tat(C22G) to produce a smaller effect (65-fold). Taken together, these results support the notion that AFF1 enhances the affinity of Tat for CycT1 more effectively than does AFF4, which in turn allows AFF1 to promote Tat activation of HIV transcription with greater efficiency.

### Reciprocal exchange of amino acids at a homologous position alters the abilities of AFF1 and AFF4 to promote Tat binding to P-TEFb and activation of HIV-1 transcription

**Figure 3.**
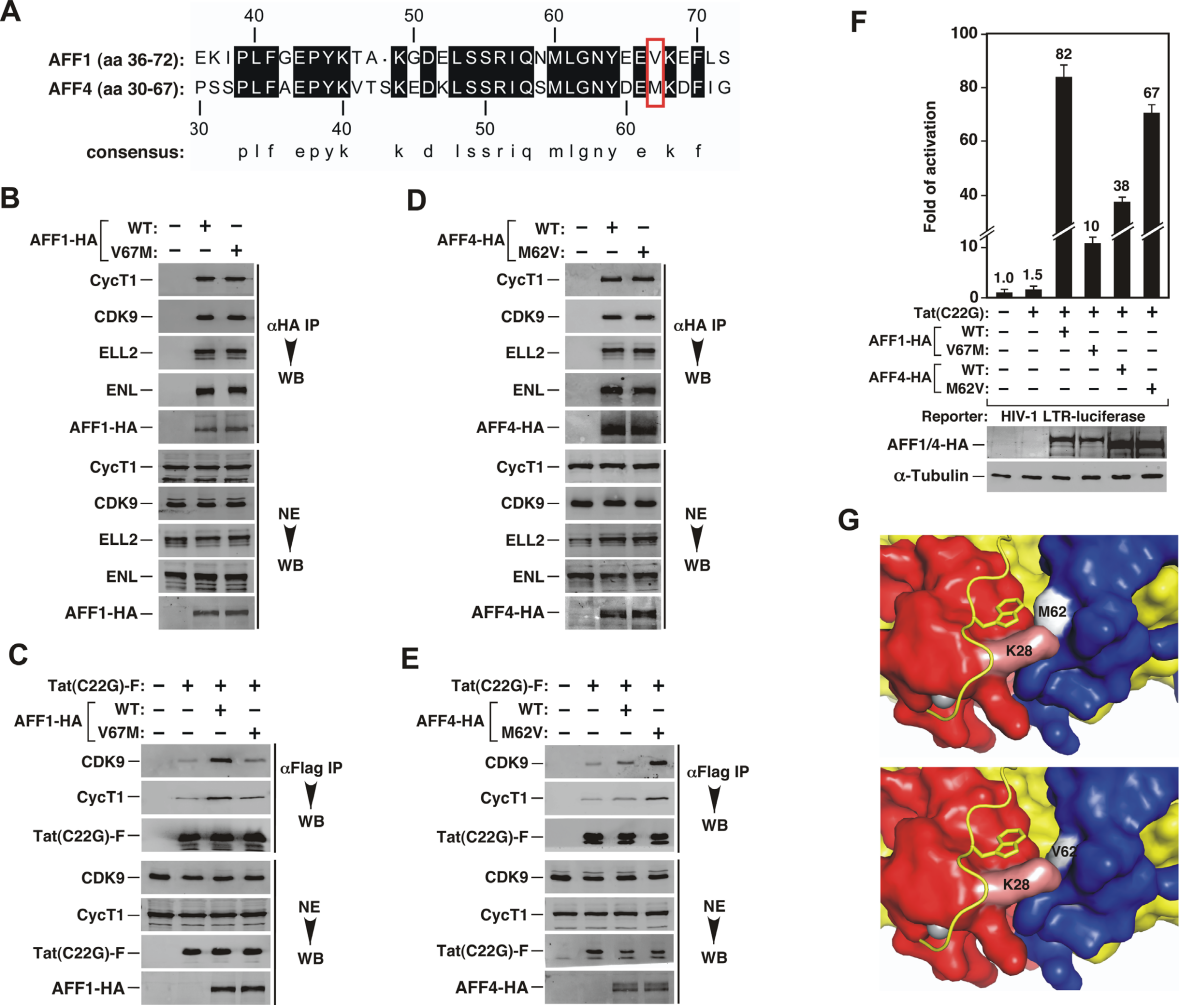
A single amino acid variation between AFF1 and AFF4 is responsible for their different abilities to promote Tat binding to P-TEFb and activation of HIV-1 transcription. (**A**) Alignment of homologous sequences encompassing the minimal CycT1-binding sequence (CBS) of AFF1 and AFF4. The dark shaded blocks indicate amino acids that are identical in the two sequences. The red box contains the residues that are believed to directly contact Tat K28. The numbers above and below the sequences denote the amino acid positions in the two AFF proeins. (**B–E**) Nuclear extracts (NEs) of HeLa cells co-transfected with the indicated expression constructs and anti-Flag immunoprecipitates (IP) derived from the NEs were examined by western blotting (WB) for the presence of the indicated proteins. (**F**) Luciferase activities were measured in extracts of NH1 cells containing the integrated HIV-1 LTR-luciferase reporter gene and expressing the indicated proteins. The activity in cells containing only the empty vectors was artificially set to 1. The error bars represent mean ± SD from three independent measurements. The cellular levels of AFF1/4-HA proteins were detected by anti-HA WB. (**G**) Top: surface representation of Tat (red)-AFF4 (blue)-CycT1 (yellow) interactions centered around Tat K28 as revealed by X-ray crystallography. K28 is forming close interactions with M62 located in WT AFF4. Bottom: model of the same structure containing the M62V point mutation in AFF4 shows a larger pocket around Tat K28 that may better accommodate the acetylated K28 side chain.

The recently solved crystal structure of P-TEFb in complex with Tat and AFF4 reveals that the AFF4 N-terminal region, which harbors the CBS (5), also makes multiple direct contacts with Tat on the surface of CycT1 (11). This observation prompted us to investigate whether sequence variations within the CBS of AFF1 and AFF4 could be responsible for the observed functional difference of the two proteins toward Tat. Notably, among the AFF4 residues that contact Tat, the structure points to M62 and F65 as particularly important (11). Since phenylalanine occupies an invariant position at 65 in AFF4 and 70 in AFF1, we focused our attention on M62 in AFF4 and its corresponding V67 in AFF1 (Figure 3A) and investigated whether this single amino acid difference could explain the different abilities of AFF1 and AFF4 in promoting Tat interaction with P-TEFb and activation of HIV-1 transcription.

To this end, we introduced the V67M point mutation in AFF1 and M62V mutation in AFF4 so that the amino acids at the corresponding positions in these two proteins were swapped reciprocally. Although AFF1 V67M was able to pull down CDK9, CycT1 and other SEC components as efficiently as WT AFF1 during immunoprecipitation (Figure 3B), it largely lost the ability to promote the interaction between Tat(C22G) and P-TEFb (Figure 3C). In comparison, AFF4 M62V, whose CBS more closely approximates that of AFF1 after the swap, gained the ability to enhance the Tat(C22G)-P-TEFb interaction (Figure 3E), although its interactions with the SEC components were essentially the same as those displayed by WT AFF4 (Figure 3D). In complete agreement with these binding results, AFF1 V67M also displayed significantly diminished ability to support the activation of HIV-1 LTR by Tat(C22G) compared to WT AFF1 (Figure 3F). In contrast, AFF4 M62V was more active than its WT counterpart in the same assay.

In Figure 3C and E, the levels of full-length AFF1, AFF4 and their point mutants in the Tat(C22G) immunoprecipitates were too low to be detected by western blotting. To solve this problem, we substituted full-length AFF1/4 with their respective N-terminal fragments (AFF1 aa 1-308 and AFF4 aa 1-300) containing the CBS in the binding assays. Comparing to the full-length proteins, these fragments were found to accumulate to a higher level, making their detection in the immunoprecipitates much easier. The data in Supplemental Figure S1 show that the AFF1/4 N-terminal fragments and their point mutants behaved exactly the same as their full-length counterparts in affecting the binding of Tat(C22G) to P-TEFb, confirming that the different abilities of AFF1/4 and their mutants to influence the Tat-P-TEFb interaction lie in their distinct N-terminal CBS regions. More importantly, the relative abundance of the WT and mutant AFF1/4 fragments that were co-precipitated with Tat(C22G) correlated precisely with that of P-TEFb bound to Tat(C22G). Thus, the two point mutations in the CBS (V67M in AFF1 and M62V in AFF4) affected the interactions of Tat with not only P-TEFb but also AFF1/4.

To better understand why a valine at position 67 in AFF1 and 62 in AFF4 was more beneficial than a methionine for AFF to promote Tat binding to P-TEFb, we compared the X-ray structure of Tat-AFF4-P-TEFb with a model where the AFF4 M62 was replaced by valine (Figure 3G). While the bulkier M62 in WT AFF4 appears to directly contact the adjacent Tat K28 residue, which is acetylated *in vivo* to enhance Tat binding to P-TEFb/TAR during HIV transactivation (36), the valine substitution at this position would create a more spacious binding pocket.

Molecular dynamics simulations of the Tat-AFF4-P-TEFb complex show the flexible Tat K28 side-chain interacting with the backbone of AFF4 as well as the Tat/TAR recognition motif (TRM) of CycT1 (M. Jacobson, personal communications). The M62V mutation in AFF4 changes the equilibrium of these interactions. In particular, K28 hydrogen bonds more frequently with the carbonyl oxygen of AFF4 E61 and has fewer interactions with the CycT1 TRM, allowing the TRM and especially the tryptophan side-chain in the TRM to adopt different conformations. Several residues in the TRM, including W258, are known to be critical for TAR interactions (37). This change in the interaction network of K28 is likely the cause for increased affinity of Tat for AFF4 M62V-P-TEFb and enhanced Tat-transactivation by AFF4 M62V.

### AFF1 facilitates Tat extraction of P-TEFb from 7SK snRNP more efficiently than does AFF4

In addition to residing in the SEC, our recent data indicate that AFF1 also associates with the 7SK snRNP through binding to CycT1 and that the AFF1-containing subpopulation of 7SK snRNP is preferentially targeted by Tat to extract P-TEFb owing to AFF1's facilitation of this process (5). In light of these observations and also the above demonstrations that AFF1 was more effective than AFF4 in enhancing the Tat-CycT1 interaction, we compared the abilities of the two AFF proteins to interact with the 7SK snRNP and assist Tat to extract P-TEFb from this complex.

**Figure 4.**
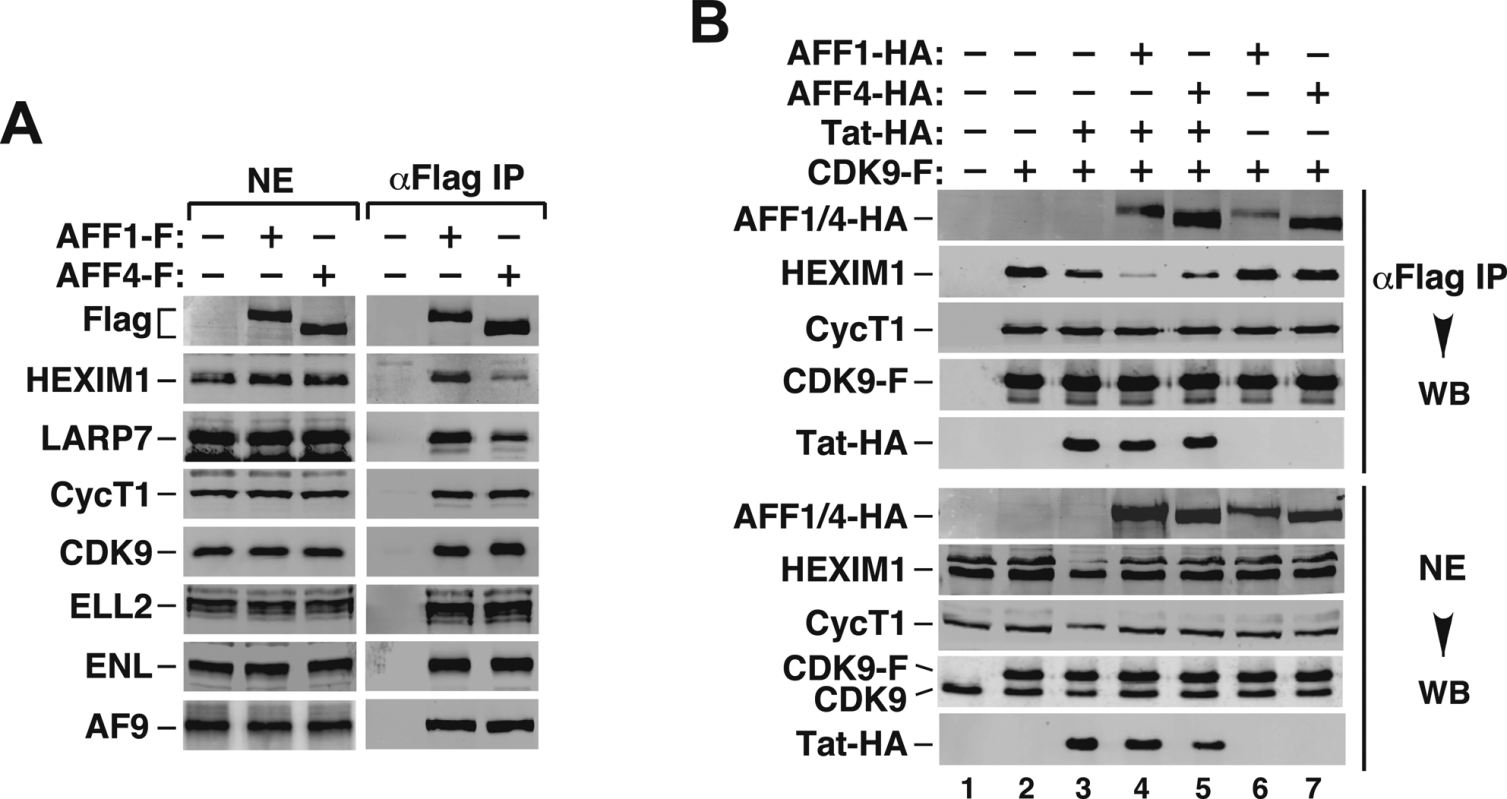
AFF1 facilitates Tat extraction of P-TEFb from 7SK snRNP more efficiently than does AFF4. (**A,B**) Nuclear extracts (NEs) prepared from HeLa cells that were transfected with the indicated expression constructs were subjected to anti-Flag immunoprecipitation (IP). The precipitates and NEs were examined by western blotting (WB) for the various proteins marked on the left.

Toward this goal, we expressed AFF1-F and AFF4-F at a similar level and performed a co-IP experiment to examine their interactions with components of the 7SK snRNP and SEC (Figure 4A). While the two proteins precipitated similar levels of the SEC subunits ELL2, ENL and AF9, AFF1-F pulled down more HEXIM1 and LARP7, the two signature 7SK snRNP components, than did AFF4-F (Figure 4A). On the other hand, although the co-expression of Tat with either AFF1 or AFF4 enhanced the disruption of 7SK snRNP more than did Tat alone as indicated by the dissociation of HEXIM1 from immunoprecipitated CDK9-F (Figure 4B, compare lane 3 with lanes 4 & 5), AFF1 was found to be more effective than AFF4 in performing this task (lanes 4 & 5). This is likely due to AFF1's stronger binding to 7SK snRNP and better accommodation of Tat than those of AFF4. Taken all these together, our data indicate that through increasing the Tat-CycT1 interaction in both the 7SK snRNP and SEC, AFF1 is a better Tat cofactor than AFF4 in promoting Tat extraction of P-TEFb from 7SK snRNP and activation of SEC-dependent HIV-1 transcription.

### AFF4- but not AFF1-containing SEC is preferentially used for heat shock-induced HSP70 expression

The data above indicate that AFF1 and AFF4 are differentially used during Tat activation of HIV-1 transcription. Together with the evidence that the two proteins exist in separate SECs, we hypothesized that the AFF1- and AFF4-SEC can have different gene target specificities. To further test this idea, we investigated the involvement of AFF1 and AFF4 during heat shock induction of HSP70 gene expression, which is another well-characterized model for studying elongation control besides the HIV-1 Tat system (1,38).

**Figure 5.**
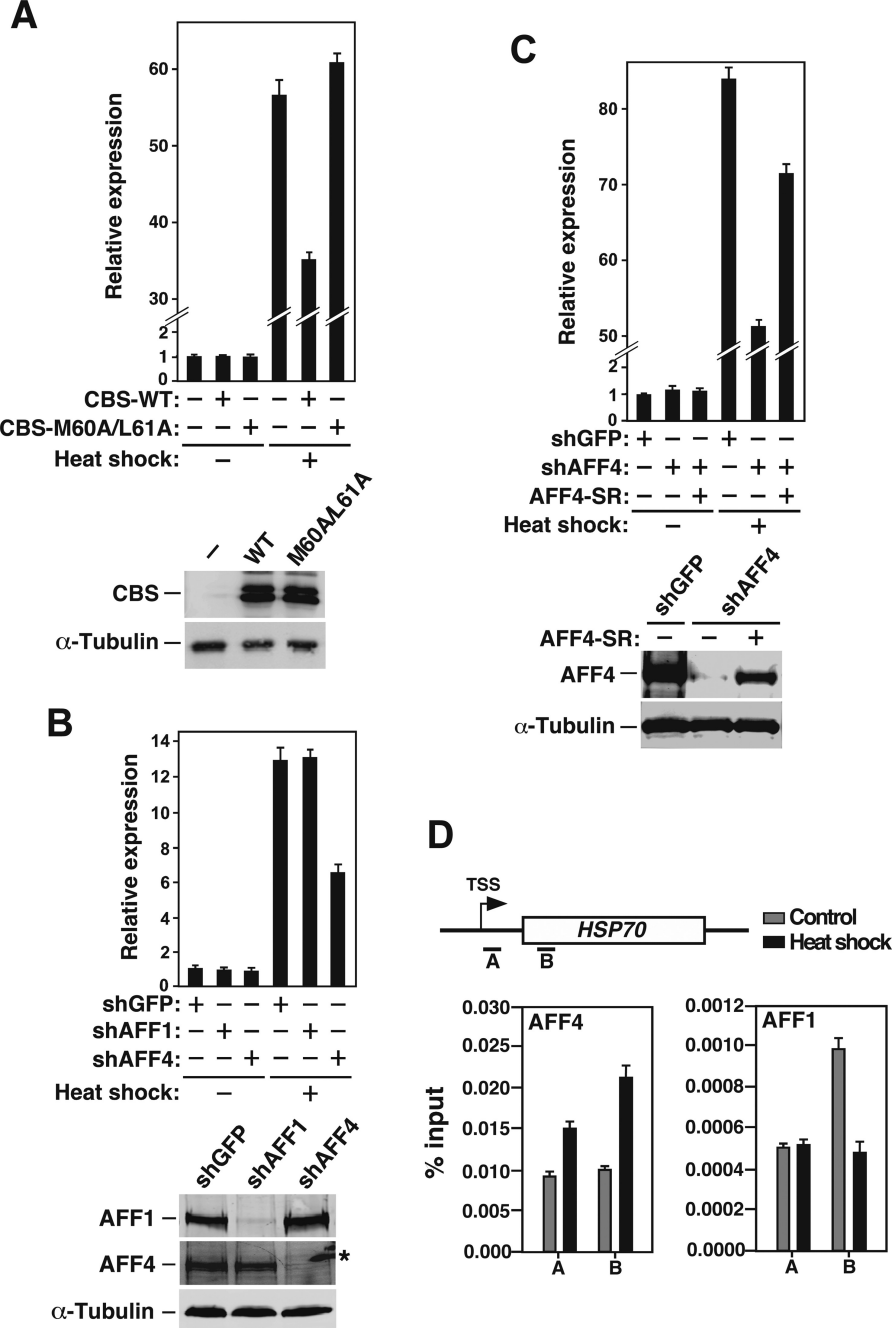
The AFF4-containing SEC is preferentially used during heat shock induction of HSP70 expression. (**A**) HeLa cells were transfected with either an empty (-) or the WT or mutant (M60A/L61A) CBS-expressing vector. Total RNAs isolated from either non-heat shocked (37°C) and heat shocked (42°C for 2 h) cells were subjected to qRT-PCR analysis to determine the levels of HSP70 mRNA relative to those of GAPDH, which was used as an internal control. The level in non-heat shocked cells transfected with an empty vector was set to 1, and the error bars represent mean ± SD from three independent measurements. The levels of WT and mutant CBS and endogenous α-tubulin were determined by western blotting (WB). (**B**) Heat shock-induced HSP70 mRNA production in cells expressing the indicated shRNAs was measured as in A. The levels of AFF1 and AFF4 in the KD cells were determined by WB, with the star (*) denotes the position of a non-specific band. (**C**) Heat shock-induced HSP70 mRNA production in cells expressing the indicated shRNAs and a shAFF4-resistent version of wild-type AFF4 (AFF4-SR) was measured as in A. The AFF4 and α-tubulin levels in transfected cells were determined by WB. (**D**) Top: genomic structure of the HSP70 locus. TSS: transcription start site. The small horizontal bars mark the positions of 2 amplicons generated by qPCR analysis of the ChIP DNA. Bottom: control or heat-treated HeLa cells were subjected to ChIP-qPCR analysis to determine the levels of AFF4 and AFF1 bound to the HSP70 locus. The signals were normalized to those of input; and the error bars represent mean ± SD from three independent experiments.

First, the dependence on a SEC for proper induction of HSP70 was examined in HeLa cells that overexpressed the AFF1 N-terminal 308 amino acids (aa) harboring the CBS. When expressed *in trans*, the CBS has been shown to dominant-negatively inhibit the SEC-dependent Tat-transactivation by preventing the full-length AFF1/4 from binding to CycT1 and recruiting ELL1/2 and ENL/AF9 into the complete SEC (5). Similar to the Tat situation, the expression of WT CBS, but not the CBS mutant M60A/L61A that fails to bind to CycT1 (5), compromised the heat shock-induced HSP70 mRNA production (Figure 5A), confirming that the SEC plays a major role in this process.

To determine the dependence on the AFF1- and AFF4-SEC for HSP70 induction, specific shRNAs (shAFF1 and shAFF4) were expressed separately in HeLa cells to knock down the expression of the two AFF proteins (Figure 5B, bottom panel). Examination by qRT-PCR indicates that the depletion of AFF4, but not AFF1, mitigated the heat shock-induced HSP70 expression (Figure 5B, top panel). The decreased HSP70 induction was not the result of an off-target effect of the shRNA as the reintroduction of a shAFF4-resistant version of cDNA expressing WT AFF4 into the KD cells rescued the heat shock-induced HSP70 expression (Figure 5C). Further analysis by ChIP indicates that heat shock increased the association of AFF4 but not AFF1 to the two 5′ positions (A & B) within the HSP70 gene locus (Figure 5D). Collectively, these data demonstrate that between the two homologous SEC complexes, the AFF4-SEC was more important for proper HSP70 induction in response to heat shock, whereas the AFF1-SEC was more potent in mediating Tat-transactivation.

### Genes regulated by AFF1- and AFF4-containing SECs are largely non-overlapping

**Figure 6.**
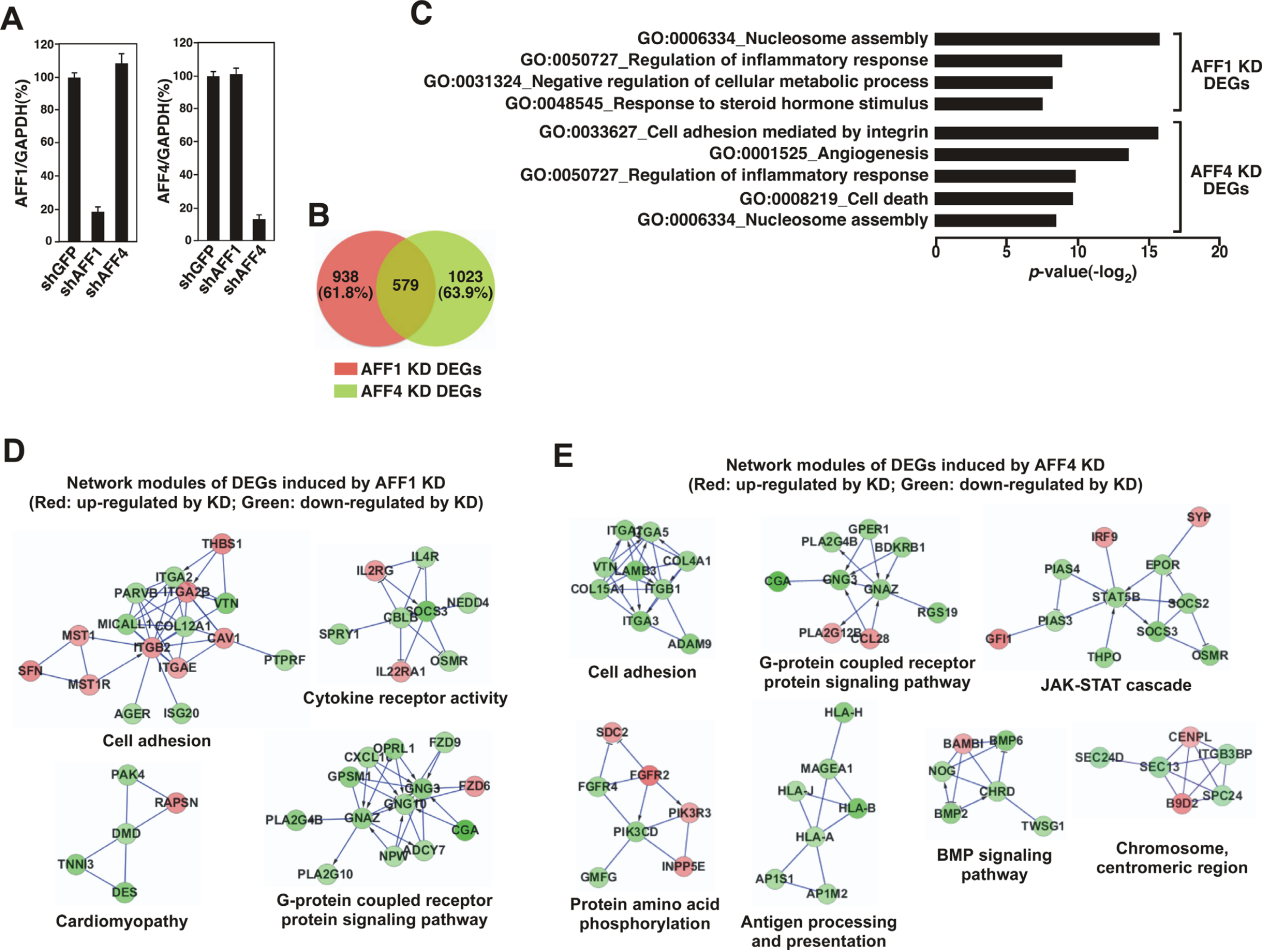
Genes regulated by AFF1- and AFF4-containing SECs are largely non-overlapping. (**A**) Total RNAs isolated from HeLa cells expressing the indicated shRNAs were analyzed by qRT-PCR to determine the levels of AFF1 (left) and AFF4 (right) mRNA relative to those of GAPDH, which was used as an internal control. (**B**) RNAs purified from the AFF1, AFF4 or the control GFP KD cells were analyzed by RNA-seq, and the numbers of differentially expressed genes (DEGs) in response to AFF1 or AFF4 KD were identified by RankProd (*P*-value ≤ 0.05) and displayed in the Venn diagram. (**C**) Gene ontology (GO) enrichment analysis of the AFF1 KD- or AFF4 KD-induced DEGs was performed using DAVID. Only the top four gene sets of the AFF KD DEGs and top five gene sets of the AFF4 KD DEGs are shown. (**D,E**) The AFF1 KD- (D) and AFF4 KD-induced DEGs (E) were mapped to a curated human functional protein interaction network and representative modules are shown. Red: genes whose expression was up-regulated by the KD; Green: genes down-regulated by KD.

To determine how the AFF1- and AFF4-SEC may function in a gene/activator-specific manner on a genome-wide scale, we expressed shAFF1 or shAFF4 in HeLa cells and performed RNA-seq to determine the downstream target genes regulated by the two types of SEC. The efficiency of KD as determined by qRT-PCR was high, with ∼80% of AFF1 and ∼90% of AFF4 depleted, respectively (Figure 6A). RNAs purified from the AFF1, AFF4 or the control GFP KD cells were prepared for single-end, high-throughput sequencing. The differentially expressed genes (DEGs) in response to AFF1 or AFF4 KD were identified by RankProd (*P*-value ≤ 0.05) (21). In total, 1517 and 1602 genes were differentially expressed in response to AFF1 and AFF4 KD, respectively (Figure 6B). Importantly, the majority of them (61.8% of the AFF1 KD- and 63.9% of the AFF4 KD-induced DEGs) were only responsive to AFF1 or AFF4 depletion, suggesting that to a large extent the AFF1- and AFF4-SEC control distinct subsets of target genes *in vivo*.

When the GO terms (39) of the DEGs were analyzed, we found that the DEGs in AFF4 KD cells were markedly enriched in cancer-related functions including cell adhesion, angiogenesis and cell death, whereas the DEGs in response to AFF1 KD were enriched in terms associated with regulation of steroid hormone response and cellular metabolic process (Figure 6C). However, both the AFF1 and AFF4 KD also caused differential expression of genes involved in inflammatory response and nucleosome assembly, suggesting that these genes are jointly regulated by the AFF1- and AFF4-SEC.

We next mapped the AFF1/4 KD-induced DEGs to a curated human functional protein interaction network to further study their relationships (23). The resulting AFF1 and AFF4 KD networks were highly modular, with the genes in similar pathways clustered together (Figure 6D and E). The genes that respond to either AFF1 or AFF4 KD share some network modules such as the G-protein coupled receptor protein signaling pathway and cell adhesion. However, many others fall into distinct, non-overlapping network modules including the ones for controlling cytokine receptor activity and cardiomyopathy in the AFF1 KD network (Figure 6D) and the ones involved the JAK-STAT cascade, protein amino acid phosphorylation, and antigen processing and presentation in the AFF4 KD network (Figure 6E).

## DISCUSSION

AFF1 and AFF4 have been proposed to exist as a dimer within a single multi-subunit SEC complex based on the observation that the two AFF proteins can pull down each other and also a complete set of the remaining SEC subunits (15,16). Although the heterodimer formation between AFF1 and AFF4 is well supported by this observation, the data presented therein cannot rule out the possibility that the dimer may in fact exist as a separate entity outside of a SEC complex. Indeed, by using the tandem affinity-purification approach described in Figure 1, we show in the current study that the AFF1-AFF4 heterodimer is not required for SEC formation and resides mostly outside of the SEC *in vivo*. Thus, an SEC can contain either AFF1 or AFF4 as its central scaffold but likely not both at the same time. This conclusion, which was obtained in engineered HEK293 cells *in vivo*, is consistent with the observations made *in vitro* demonstrating a mutually exclusive existence of AFF1 and AFF4 in reconstituted SEC complexes (28).

Previously, we have reported that the highly homologous ENL and AF9 proteins also exist in separate SECs (9). Furthermore, there is no evidence indicating that the homologous ELL1, ELL2 and ELL3 can ever form dimers (16), and thus they most likely reside in different SECs as well. Notably, ELL1 has also been found as a component of the so-called Little Elongation Complex that is involved in small nuclear RNA (snRNA) gene expression (40), while ELL3 appears to be specifically selected to prime gene expression by marking enhancers in ES cells (41). Finally, CycT1 and its close homologues CycT2a and T2b are also not known to exist in a single high-order complex. Together, these observations strongly support the notion that the SEC is in fact a large family of closely related complexes made possible by different combinations among groups of homologous proteins that are all bona fide SEC subunits but do not exist in a single mega complex all at once. This arrangement has the benefit of using a relatively small number of individual components to create a much bigger collection of functional assemblies, which in turn can significantly increase the regulatory diversity and gene-control options during SEC-mediated activation of transcription of diverse genes.

In support of the idea that members of the SEC family of complexes can have gene/activator-specific functions, our current study shows that HIV Tat strongly prefers the AFF1-SEC over the AFF4-SEC in activation of viral transcription. The preference has been traced to a single amino acid difference located within the N-terminal CBS region between the two AFF proteins. The crystal structure shows that AFF4 residue 62 is at the nexus of the interactions between AFF4, Tat and CycT1 (11). Replacing the bulky methionine at this position with the smaller valine results in a larger binding pocket for Tat K28, an amino acid that is strictly conserved and exquisitely regulated by reversible acetylation during Tat-transactivation (3,36). Molecular modeling showed that the interaction network of Tat K28 is different within the Tat-AFF4-P-TEFb complex upon the M62V mutation, with K28 making tighter interactions with the AFF4 backbone and fewer interactions with the CycT1 TRM. These changes provide a likely explanation for the increased affinity of Tat for AFF4 M62V-P-TEFb (Figure 3E and Supplemental Figure S1) and enhanced Tat-transactivation by the M62V mutant AFF4 compared to the WT protein (Figure 3E). Unlike AFF4, AFF1 naturally has a valine at the corresponding position, which explains why AFF1 was found to increase the Tat-CycT1 interaction more effectively than did AFF4 (Figure 2D).

We have previously shown that ELL2 is strongly preferred over ELL1 as a key SEC subunit to support efficient Tat-transactivation (7), although the molecular basis of this preference is still unknown. Between the homologous ENL and AF9 pair, the SECs containing the latter protein were found to be about 45% more active in mediating Tat-activated HIV transcription (9). Finally, among the SEC subunits CycT1, CycT2a and CycT2b, CycT1 is the only one that is capable of supporting Tat-transactivation due to its unique TRM that is essential for establishing a stable Tat-P-TEFb/SEC complex on HIV TAR RNA (2,3). It is therefore highly likely that among all the members of the SEC family, Tat specifically selects the ones containing AFF1, ELL2, CycT1 and AF9 for maximal activation of HIV-1 transcriptional elongation.

In addition to AFF1 and AFF4, the other two AF4/FMR2 family members, AFF2 and AFF3, have also been shown to exist in SEC-like complexes termed SEC-L2 and SEC-L3, respectively (42). Although SECs and SEC-L2 and -L3 have similar kinase activities toward the Pol II CTD, genome-wide analysis suggests that these complexes regulate different subsets of genes in cells (42). Importantly, the current study has extended this notion to even members within the SEC family by showing that the AFF1-SEC and AFF4-SEC can also have different gene target specificities on a global scale. While our analyses have revealed the molecular basis underlying this difference in the case of Tat-transactivation, little is known about how other activators/genes select one type of SEC over others for their proper transactivation. For example, during heat shock response, it is unclear how the AFF4-SEC becomes specifically selected to bind to the HSP70 gene promoter for induction of expression (Figure 5D) and whether the HSF transcription factors (38) may play a key role in this process. Future studies are necessary to answer these questions and elucidate the molecular mechanism used by cells to differentially use members of the SEC family to not only achieve gene target specificity but also enhance regulatory diversity during transcriptional activation of diverse genes.

## SUPPLEMENTARY DATA

Supplementary Data are available at NAR Online.

SUPPLEMENTARY DATA
