# Supplementary material for: Gene target specificity of the Super Elongation Complex (SEC) family: how HIV-1 Tat employs selected SEC members to activate viral transcription
Source: Nucleic Acids Res. 2015 May 24;43(12):5868–79. doi: 10.1093/nar/gkv541 (PMC4499153; doi:10.1093/nar/gkv541)
Supplement: SUPPLEMENTARY DATA [file supp_43_12_5868__index.html]

Gene target specificity of the Super Elongation Complex (SEC) family: how HIV-1 Tat employs selected SEC members to activate viral transcription — Gene target specificity of the Super Elongation Complex (SEC) family: how HIV-1 Tat employs selected SEC members to activate viral transcription — SUPPLEMENTARY DATA 

# Gene target specificity of the Super Elongation Complex (SEC) family: how HIV-1 Tat employs selected SEC members to activate viral transcription

## SUPPLEMENTARY DATA

- SUPPLEMENTARY DATA
