# Supplementary material for: Gene target specificity of the Super Elongation Complex (SEC) family: how HIV-1 Tat employs selected SEC members to activate viral transcription
Source: Nucleic Acids Res. 2015 May 24;43(12):5868–79. doi: 10.1093/nar/gkv541 (PMC4499153; doi:10.1093/nar/gkv541)

Inventory of Supplemental Information

This document contains Supplemental Figure S1 and legend that pertain to main Figure 3.

Title: Gene target specificity of the Super Elongation Complex (SEC) family: How HIV-1 Tat employs selected SEC members to activate viral transcription

By Huasong Lu, Zichong Li, Wei Zhang, Ursula Schulze-Gahmen, Yuhua Xue, and Qiang Zhou

Supplemental Figure and Legend

Figure S1 (related to main Figure 3). Point mutations in the CBS regions of AFF1 and AFF4 affect the interactions of Tat with not only P-TEFb but also AFF1/4 themselves. A & B. HeLa cells were co-transfected with the indicated expression constructs. Nuclear extracts (NE) from the transfected cells as well as anti-Flag immunoprecipitates (IP) derived from the NE were examined by Western blotting (WB) for the presence of the labeled proteins.

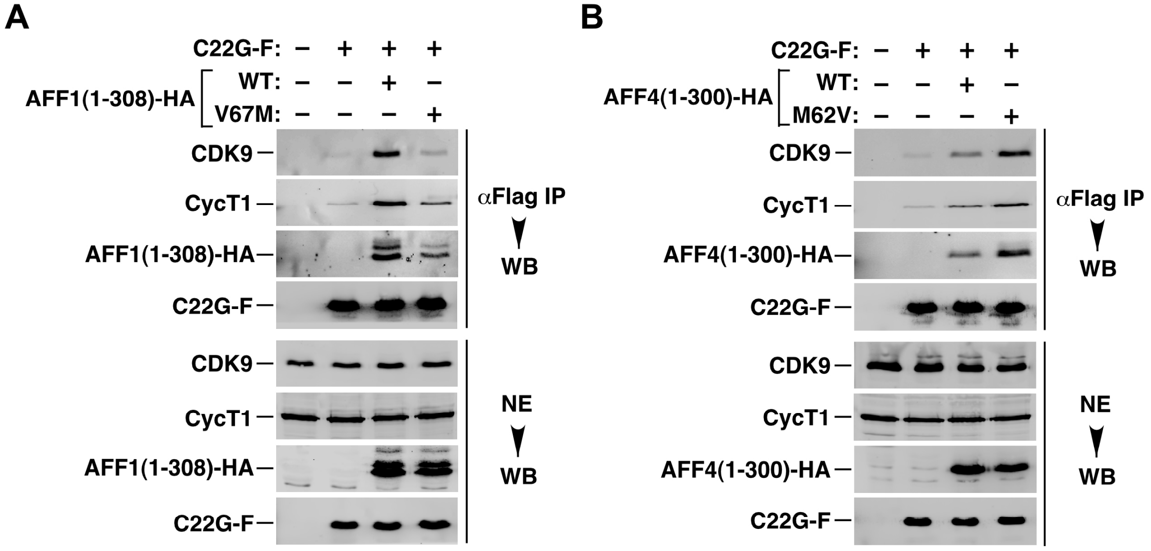

Supplement: SUPPLEMENTARY DATA [file supp_gkv541_nar-01001-a-2015-File008.pdf]
